# Factors of pre-existing low muscle mass and its relationship with mortality in critically ill patients: a sex-specific analysis

**DOI:** 10.3389/fmed.2026.1903468

**Published:** 2026-08-19

**Authors:** Weixia Yu, Shiping Wang, Haifang Wang, Jianzheng Cai, Yingying Zhang

**Affiliations:** 1 Department of Intensive Care Medicine, The First Affiliated Hospital of Soochow University, Suzhou, Jiangsu, China; 2 Department of Nursing, The First Affiliated Hospital of Soochow University, Suzhou, Jiangsu, China; 3 Department of Chemotherapy, The First Affiliated Hospital of Soochow University, Suzhou, Jiangsu, China

**Keywords:** computed tomography, critical care nutrition, critical illness, factors, low muscle mass, skeletal muscle mass

## Abstract

**Objectives:**

To investigate the factors, and mortality associations of pre-existing low muscle mass in ICU (intensive care unit) patients, with emphasis on sex-stratified analysis.

**Methods:**

This cross-sectional study included 760 adult ICU patients (513 males, 247 females). Pre-existing low muscle mass was defined using sex-specific cutoffs based on CT. Multivariable logistic regression, restricted cubic spline (RCS) analysis were used to identify the factors associated with pre-existing low muscle mass. Cox proportional hazards models were applied to assess the association between pre-existing low muscle mass and 28-day mortality, adjusting for potential confounders.

**Results:**

Among 760 ICU patients, pre-existing low muscle mass prevalence was 34.0% (males 41.3% vs. females 19.8%). Higher BMI was inversely associated with pre-existing low muscle mass in both sexes (males: aOR = 0.73, *P* < 0.001; females: aOR = 0.82, *P* < 0.001). High nutritional risk (NRS 2002 ≥ 4) and high comorbidity burden (aCCI ≥ 4) were independently associated with higher odds of pre-existing low muscle mass. Additioinally, RCS analysis revealed linear inverse BMI–pre-existing low muscle mass associations. Exploratory analysis suggested that the inverse association may plateau at higher BMI levels; however, these threshold values are hypothesis-generating and require prospective validation. In males, pre-existing low muscle mass was associated with higher 28-day mortality in unadjusted models, but these associations were attenuated after adjusting for confounders.

**Conclusions:**

BMI, nutritional risk, and comorbidity burden are key cross-sectional factors, which support early risk stratification using BMI, NRS 2002, and aCCI upon ICU admission. The exploratory plateau values are hypothesis-generating and need prospective validation.

## 1 Introduction

The number of critically ill patients admitted to the intensive care unit (ICU) continues to rise globally. In Poland, ICU admission rates increased from 1.6 per 1000 in 2012 to 2.2 per 1000 in 2021, with a male predominance of 56.6 % compared to 43.4 % for females (1). Similarly, a nationwide Korean analysis reported an age-standardized ICU admission rate of 744.6 per 100,000 person-years during 2009–2014 (2). With continuous advancements in critical care technology, an increasing number of critically ill patients are surviving their acute illness (3, 4). Among the numerous factors influencing patient survival, muscle mass has emerged as an important predictor of adverse clinical outcomes (5). Previous studies have demonstrated that acute muscle mass loss can occur shortly after ICU admission (6, 7). More recently, pre-existing low muscle mass at the time of ICU admission has garnered significant attention from researchers and clinicians (8–10).

Pre-existing low muscle mass, also known as low muscle mass at ICU admission, is highly prevalent in critically ill patients. A meta-analysis by Jiang et al. (11) involving 3,582 patients on mechanical ventilation, revealed a 43% incidence of pre-existing sarcopenia. Also, the prevalent of pre-existing low muscle mass in critically ill patients with hematologic malignancies was 64.9% at the ICU admission (12). The vulnerability of patients with low baseline muscle mass at the time of ICU admission to adverse outcomes has also been underscored. Specifically, patients with pre-existing low muscle mass admitted to the ICU tended to have poorer short-term outcomes, such as higher mortality rates, longer durations on mechanical ventilation, extended ICU stays, and prolonged hospitalizations (13–16). Furthermore, ICU survivors with low muscle mass at admission showed lower grip strength, physical dysfunction, and a higher 1-year mortality (17, 18). Hence, early identification of individuals with pre-existing low muscle mass is significantly necessary.

Although existing studies have investigated the incidence of pre-existing low muscle mass, as well as its short-and long-term outcomes, the cross-sectional correlates of this condition at ICU admission remain inadequately characterized. This delays early identification of these populations and hinders timely measures to prevent further muscle loss and negative outcomes. Therefore, identifying correlates of individuals with low muscle mass upon admission to the ICU is important for ICU teams to accurately assess baseline risk and plan preventing nutrition and rehabilitation strategies. Meanwhile, muscle loss exhibits sex differences between males and females (19). Therefore, research on its cross-sectional correlates should be conducted based on sex-specific considerations.

This study aims to investigate the sex-specific cross-sectional correlates of pre-existing low muscle mass in critically ill patients admitted to the ICU, and to explore their association with 28-day mortality.

## 2 Materials and methods

### 2.1 Study design

This study utilized a cross-sectional design to comprehensively investigate the correlates of low muscle mass and its association with 28-day mortality. The reporting followed the Strengthening the Reporting of Observational Studies in Epidemiology (STROBE) checklist from the EQUATOR Network. Detailed in Supplementary Table S1.

### 2.2 Setting and sample

Patients hospitalized between October 2024 and November 2025 at the First Affiliated Hospital of Soochow University were screened for eligibility. Inclusion criteria were all adults (≥18 years) admitted to an ICU, had available abdominal computed tomography (CT) scans performed within 48 h during the ICU admission. We excluded patients who had missing data on 28 days mortality, survival time. Participants without laboratory indicators were also excluded.

According to the estimation of sample size for cross-sectional and prevalence studies, the sample size is determined by the following formula (20), where *N* is the sample size, *Z* represents the confidence level, *P* is the anticipated prevalence, and d is the precision. Research by Meyer et al. (21) Revealed that the prevalence of CT-defined sarcopenia at ICU admission was 0.16. Thus, the sample size for a precision of 0.05 and *Z* = 1.96 was 206 participants.


$$\begin{array}{l} N=\frac{Z_{1-\partial /2}^{2}\times P\left(1-P\right)}{d^{2}} \end{array}$$

### 2.3 Data collection

We collected the following baseline clinical data from electronic medical records, including age, sex, body mass index (BMI), acute physiology and chronic health evaluation (APACHE) II score, sequential organ failure assessment (SOFA) score, nutrition risk screening (NRS) 2,002 score, major disease diagnosis classification, age-adjusted Charlson comorbidity index (aCCI), comorbidities (high blood pressure, HBP; coronary heart disease, CHD), albumin, prealbumin, lymphocyte count, monocyte count, neutrophil count, platelet-to-lymphocyte ratio (PLR), systemic inflammatory response index (SIRI = neutrophil count × monocyte count/lymphocyte count), and prognostic nutrition index (PNI = 10 × albumin + 0.005 × lymphocyte count) within 24 h admission to ICU, skeletal muscle area (SMA) and skeletal muscle inedx (SMI) within 48 h admission to ICU. We also collected 28 days mortality.

### 2.4 Muscle mass assessment

**Figure 1:**
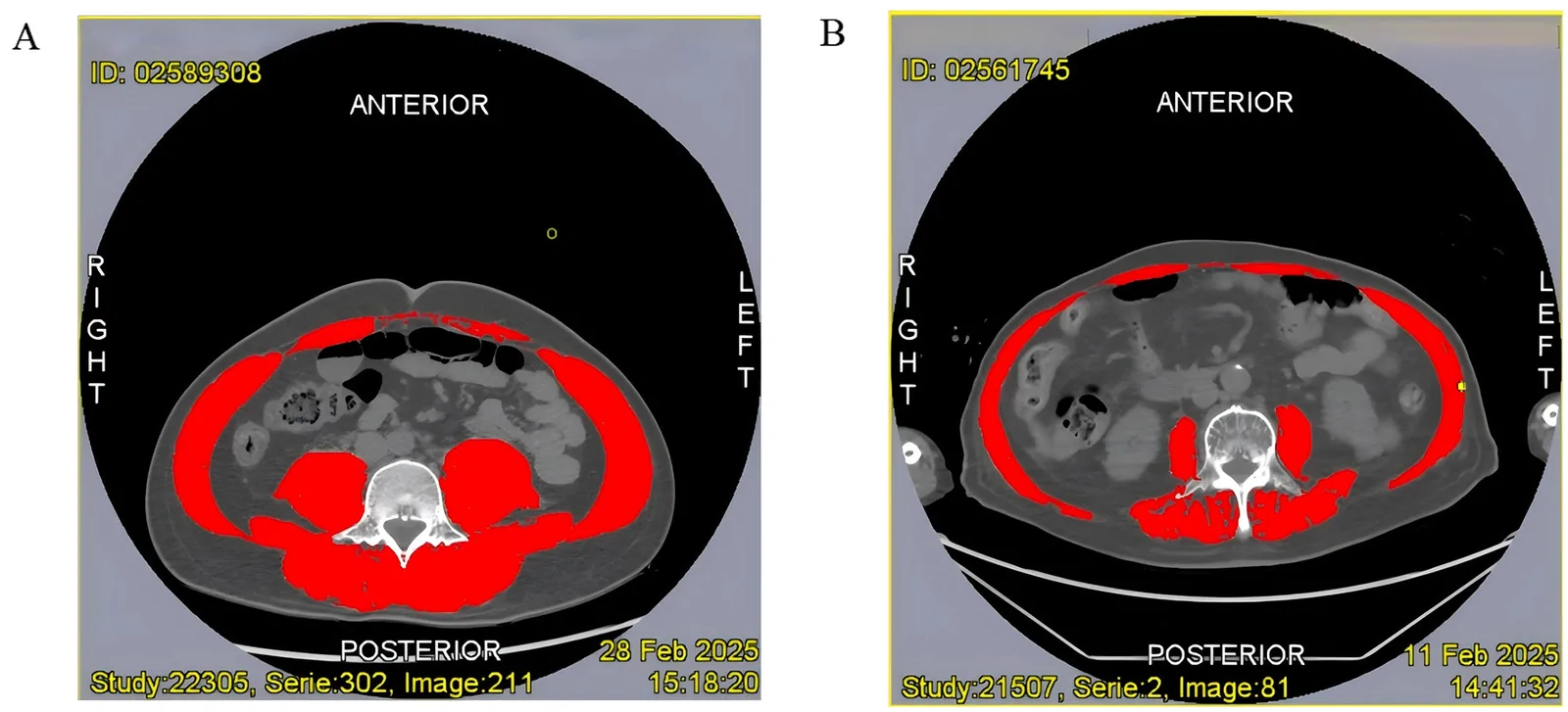
Skeletal muscle area on L3 axial slice. Measuring skeletal muscle area on L3 axial slice Computer-Tomography (CT) Image was measured by the SliceOmatic software (5.0 Rev-9). Radio density thresholds were selected to correlate with those of skeletal muscle, with lower and upper thresholds of −29 and 150 Hounsfield units, respectively. **(A)** normal muscle mass; **(B)** low muscle mass.

Abdominal CT images taken at the L3 vertebral level should be the reference for defining muscle status (22), which were obtained from the Picture Archiving and Communication System (PACS), in Digital Imaging and Communication in Medicine (DICOM) format. Abdominal CT imaging performed within 48 h of the date of hospitalization were used to defined preexisting low muscle mass. Skeletal muscle area was measured manually by using the SliceOmatic software (5.0 Rev-9), as illustrated in Figure 1. The L3-skeletal muscle inedx was calculated as follows: the skeletal muscle area at the L3 level divided by the square of the height (cm^2^/m^2^). Specifically, the sex-specific pre-existing low muscle mass was defined as lower to the skeletal muscle inedx (44.77 cm^2^/m^2^ for male patients and 32.50 cm^2^/m^2^ for female patients), as described in previous Chinese population (23).

### 2.5 Ethical approval

This study was conducted in accordance with ethical guideline. The ethical board of the First Affiliated Hospital of Soochow University reviewed and approved the study protocol (No.2024577). Informed consent was obtained directly from patients when they possessed decision-making capacity. When patients lacked capacity at the time of ICU admission, written informed consent was obtained from the patient's legal guardian or next of kin.

### 2.6 Statistic

Data analysis used R 4.5.2 (R Foundation, Boston, MA, USA). Baseline characteristics were presented by muscle mass status. Normality for continuous variables was tested using the Shapiro-Wilk test. Normally distributed data were described as means and standard deviations; nonnormally distributed data as medians and interquartile ranges. Categorical data were presented as numbers and percentages. The differences in the continuous and categorical variables between different sex and muscle mass status were assessed using Wilcoxon's rank-sum test and Chi-square test/Fisher's exact test, respectively.

Multicollinearity was assessed using variance inflation factor (VIF). Logistic regression was used to identify cross-sectional correlates of pre-existing low muscle mass. Guided by the principles of the purposeful variable selection framework, variables with univariate significant difference were included in the multivariable model (24). Results were presented as odds ratios (ORs) and their 95% confidence intervals (95%CIs) for the final regression model. Restricted cubic splines (RCS, knots at 10th, 50th, 90th percentiles) based on logistic regression explored descriptive nonlinear associations of significant factors (*P* < 0.1 or ~0.1) with pre-existing low muscle mass, with adjustment for confounders; non-linearity was tested by likelihood ratio test (*P* < 0.1 or ~0.1 indicating non-linearity).

Cox proportional hazard models analyzed associations between pre-existing low muscle mass and 28-day mortality. Schoenfeld residuals was performed to check the proportional hazards assumption. Two Cox models were fit: model 1 (crude) was unadjusted, while Model 2 (adjusted) adjusted for age, BMI classification, aCCI risk stratification, major disease diagnosis, SOFA score, APACHE II score, and prognostic nutrition index. Results were expressed as hazard ratios (HRs) with 95% CIs.

Given the exploratory nature of this cross-sectional study and the limited sample size in certain subgroups (particularly females, *n* = 247 with only 49 cases), a threshold of *P* < 0.10 was adopted throughout to balance the risk of Type II error against the risk of Type I error, consistent with conventions in exploratory epidemiological analyses (25).

## 3 Results

### 3.1 Patient characteristics

**Figure 2:**
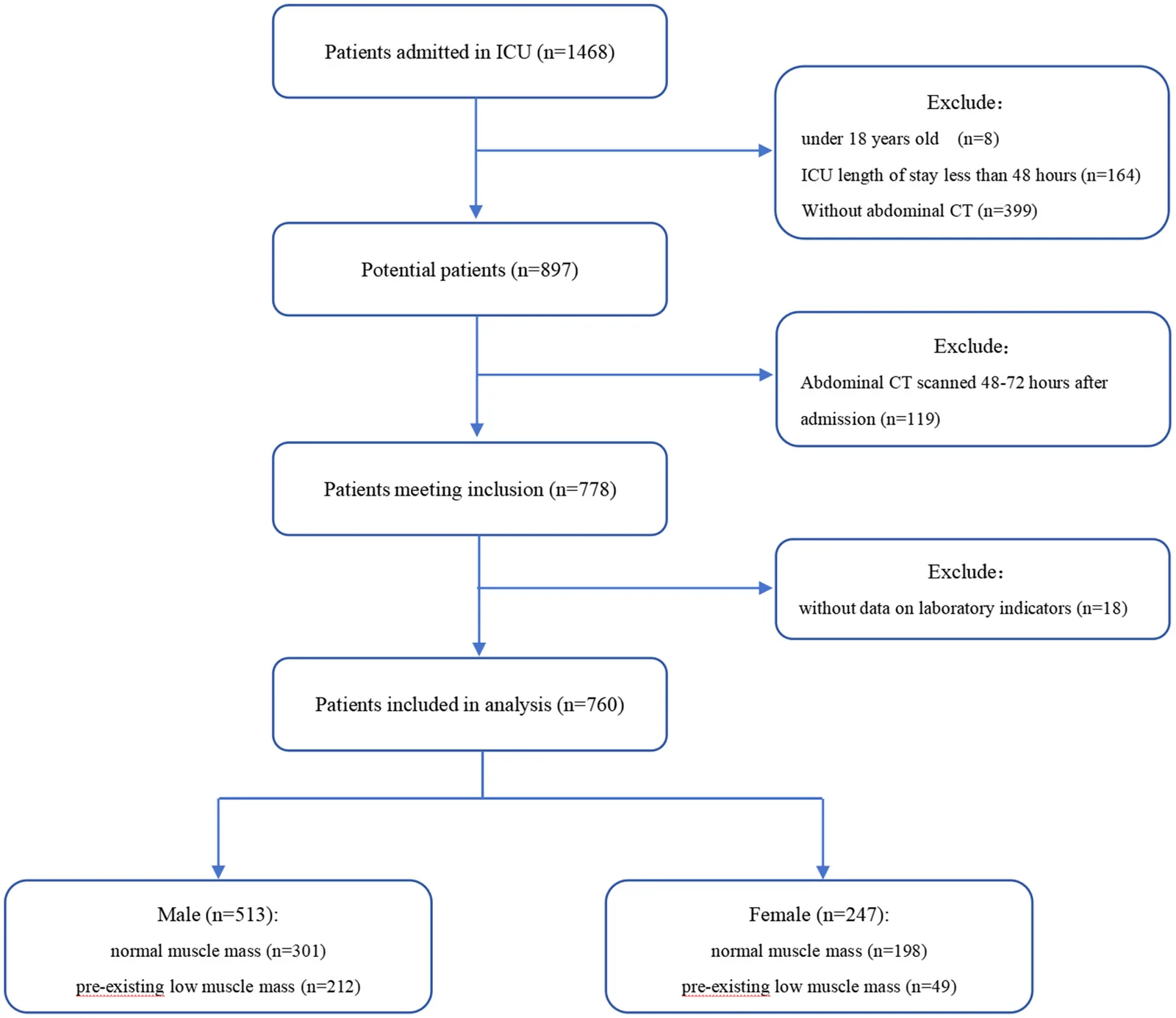
Flow diagram of patient selection.

Of 1468 ICU admissions, 760 patients were included (513 males, 247 females). Among them, 212 males and 49 females had pre-existing low muscle mass. Patient selection is shown in Figure 2.

Table 1 compares baseline characteristics by muscle mass status and sex. In both sexes, the pre-existing low muscle mass group had higher age, APACHE II, SOFA, NRS 2002, and aCCI (*P* < 0.001), and lower BMI, SMA, and SMI (*P* < 0.001). In males, 28-day mortality (13.7% vs. 5.3%, *P* = 0.002) were higher in the pre-existing low muscle mass group.

**Table 1:** Patient characteristics at baseline.

Characteristics	Male (*N* = 513)			Female (*N* = 247)		
	Normal muscle mass (*N* = 301)	Pre-existing low muscle mass (*N* = 212)	*P*	Normal muscle mass (*N* = 198)	Pre-existing low muscle mass (*N* = 49)	*P*
Age (years)	54.0 [37.0, 65.0]	70.0 [58.0, 77.0]	**< 0.001**	61.0 [47.0, 73.0]	70.0 [58.0, 77.0]	**0.003**
BMI (kg/m^2^)	24.5 [22.5, 27.3]	21.1 [19.3, 23.4]	**< 0.001**	23.5 [20.8, 25.7]	20.6 [18.7, 22.6]	**< 0.001**
BMI classification			**< 0.001**			**0.001**
Underweight	8 (2.66%)	42 (19.8%)		11 (5.56%)	11 (22.4%)	
Normal	108 (35.9%)	124 (58.5%)		99 (50.0%)	28 (57.1%)	
Overweight	133 (44.2%)	44 (20.8%)		67 (33.8%)	7 (14.3%)	
Obese	52 (17.3%)	2 (0.94%)		21 (10.6%)	3 (6.12%)	
APACHE II score (points)	16.0 [12.0, 23.0]	20.0 [14.0, 26.0]	**< 0.001**	18.0 [12.0, 24.0]	22.0 [17.0, 28.00]	**0.002**
SOFA score (points)	4.0 [2.0, 7.0]	5.0 [3.0, 8.0]	**0.002**	4.0 [2.3, 7.0]	5.0 [3.0, 7.0]	**0.056**
NRS 2002 score (points)	3.0 [3.0, 4.0]	4.0 [3.0, 5.0]	**< 0.001**	3.0 [3.0, 4.0]	4.0 [3.0, 6.0]	**< 0.001**
Nutrition risk stratification			**< 0.001**			**< 0.001**
Low risk (NRS 2002 ≤ 3points)	209 (69.4%)	62 (29.2%)		111 (56.1%)	13 (26.5%)	
High risk (NRS 2002 ≥ 4points)	92 (30.6%)	150 (70.8%)		87 (43.9%)	36 (73.5%)	
Major disease diagnosis classification			**< 0.001**			**0.010** ^*^
Wound	103 (34.2%)	35 (16.5%)		49 (24.7%)	7 (14.3%)	
Respiratory system disease	24 (7.97%)	32 (15.1%)		22 (11.1%)	14 (28.6%)	
Sepsis	23 (7.64%)	27 (12.7%)		20 (10.1%)	7 (14.3%)	
Neurological diseases	29 (9.63%)	27 (12.7%)		20 (10.1%)	7 (14.3%)	
Digestive system disease	80 (26.6%)	63 (29.7%)		48 (24.2%)	4 (8.16%)	
cardiovascular disease	14 (4.65%)	9 (4.25%)		16 (8.08%)	4 (8.16%)	
kidney/blood/metabolic diseases/other	28 (9.30%)	19 (8.96%)		23 (11.6%)	6 (12.2%)	
aCCI (points)	3.0 (1.0, 6.0)	8.0 (5.0, 10.)	**< 0.001**	5.0 (3.0, 8.0)	8.0 (5.0, 9.0)	**< 0.001**
aCCI risk stratification			**< 0.001**			**0.002**
Low to medium risk (aCCI ≤ 3points)	170 (56.5%)	35 (16.5%)		73 (36.9%)	6 (12.2%)	
high risk (aCCI ≥ 4points)	131 (43.5%)	177 (83.5%)		125 (63.1%)	43 (87.8%)	
Combined HBP			0.357			0.211
No	181 (60.1%)	118 (55.7%)		119 (60.1%)	24 (49.0%)	
Yes	120 (39.9%)	94 (44.3%)		79 (39.9%)	25 (51.0%)	
Combined CHD			**0.002**			**0.114**
No	294 (97.7%)	193 (91.0%)		192 (97.0%)	45 (91.8%)	
Yes	7 (2.33%)	19 (8.96%)		6 (3.03%)	4 (8.16%)	
Albumin stratification			**0.003**			0.633
Low level (< 30g/L)	216 (71.8%)	177 (83.5%)		162 (81.8%)	38 (77.6%)	
Normal level (≥30g/L)	85 (28.2%)	35 (16.5%)		36 (18.2%)	11 (22.4%)	
Prealbumin (mg/L)	147.0 [106.0, 195.0]	116.0 [72.6, 167.0]	**< 0.001**	113.0 [83.5, 151.0]	137.0 [75.4, 163.0]	0.240
Lymphocyte Count (10^9^/L)	0.8 [0.5, 1.1]	0.6 [0.4, 0.9]	**0.001**	0.8 [0.5, 1.2]	0.6 [0.4, 0.9]	**0.071**
Monocyte Count (10^9^/L)	0.6 [0.4, 0.8]	0.5 [0.3, 0.7]	**< 0.001**	0.5 [0.3, 0.7]	0.4 [0.2, 0.6]	**0.134**
Neutrophil Count (10^9^/L)	9.8 [6.9, 13.2]	8.6 [6.0, 12.5]	**0.061**	9.7 [6.5, 13.1]	9.8 [5.9, 12.0]	0.492
PLR	209.0 [130.0, 331.0]	235.0 [149.0, 404.0]	**0.033**	214.0 [126.0, 342.0]	255.0 [153.0, 417.0]	0.225
Systemic inflammatory response index	7.5 [3.6, 13.1]	5.8 [2.9, 11.1]	**0.077**	5.3 [2.6, 11.4]	4.4 [2.3, 10.4]	0.703
Prognostic Nutrition index	322.0 [286.0, 358.0]	312.00 [275.0, 339.0]	**0.019**	309.0 [275.0, 334.0]	317.0 [300.0, 346.0]	**0.070**
SMA (cm^2^)	159.0 [144.0, 174.0]	113.00 [99.5, 126.0]	**< 0.001**	101.0 [92.3, 113.0]	73.7 [64.8, 80.6]	**< 0.001**
SMI (cm^2^/m^2^)	53.2 [49.2, 58.4]	39.00 [35.3, 42.3]	**< 0.001**	40.3 [37.4, 45.3]	29.2 [25.6, 31.1]	**< 0.001**
Death within 28 days			**0.002**			0.223
No	285 (94.7%)	183 (86.3%)		180 (90.9%)	41 (83.7%)	
Yes	16 (5.3%)	29 (13.7%)		18 (9.09%)	8 (16.3%)	
Death in ICU			**< 0.001**			0.173
No	284 (94.4%)	179 (84.4%)		178 (89.9%)	40 (81.6%)	
Yes	17 (5.7%)	33 (15.6%)		20 (10.1%)	9 (18.4%)	

Continuous variables are presented as median [IQR]. Categorical variables are presented as counts (%). In bold are indicated *P* values considered as statistically significant (*P* < 0.1 or approximately 0.1). ^*^Fisher's exact test. BMI, body mass index; APACHE II, acute physiology and chronic health evaluation II; SOFA, sequential organ failure assessment; NRS 2002, nutrition risk screening 2002; aCCI, age-adjusted Charlson comorbidity index; HBP, high blood pressure; CHD, coronary heart disease; PLR, platelet-to-lymphocyte ratio.

### 3.2 Prevalence of pre-existing low muscle mass

**Figure 3:**
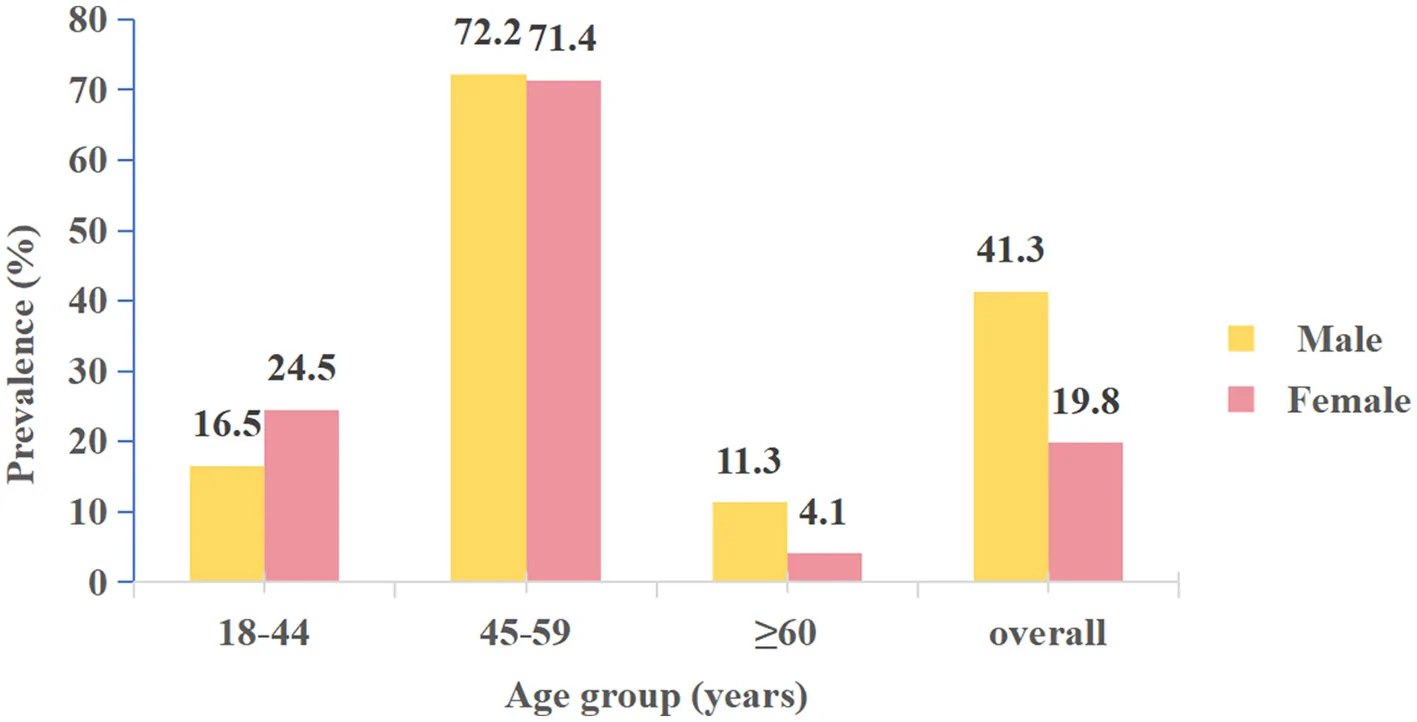
Prevalence of pre-existing low muscle mass by sex and age group.

Overall prevalence was 34.0% (41.3% in males, 19.8% in females). By age group (18–44, 45–59, ≥60 years), the prevalence was 16.5/24.5%, 72.2/71.4%, and 11.3/4.1% in males/females, respectively (Figure 3).

### 3.3 Factors associated with pre-existing low muscle mass

Multicollinearity diagnostics using VIF indicated no significant collinearity among the included variables (all VIF < 5) (Supplementary Table S2). Table 2 shows the results of multivariable logistic regression. In males, higher BMI (aOR = 0.73, *P* < 0.001) and higher monocyte count (aOR = 0.38, *P* = 0.04) were associated with lower odds of pre-existing low muscle mass; NRS 2002 ≥ 4 (aOR = 2.00, *P* = 0.015), aCCI ≥ 4 (aOR = 2.54, *P* = 0.034), and combined CHD (aOR = 2.30, *P* = 0.105—a non-significant trend) were associated with higher odds.

**Table 2:** Logistic regression associated with pre-existing low muscle mass.

Characteristics	Male		Female	
	OR (95%CI)	*P*	OR (95%CI)	*P*
Age (years)	1.00 (0.98–1.02)	0.992	1.00 (0.96–1.04)	0.933
BMI (kg/m^2^)	0.73 (0.67–0.79)	**< 0.001**	0.82 (0.73–0.92)	**< 0.001**
APACHE II score (points)	0.99 (0.96–1.03)	0.748	1.02 (0.96–1.08)	0.589
15.6-7.4,-1.3498ptSOFA score (points)	0.99 (0.91–1.07)	0.755	0.95 (0.83–1.08)	0.412
Nutrition risk stratification				
Low risk (NRS 2002 ≤ 3points)	Reference		Reference	
15.6-7.4,-1.3498ptHigh risk (NRS 2002≥4points)	2.00 (1.14–3.50)	**0.015**	2.25 (0.88–5.77)	**0.092**
Major disease diagnosis classification				
Wound	Reference		Reference	
Respiratory system disease	1.74 (0.75–4.07)	0.199	2.22 (0.64–7.76)	0.209
Sepsis	1.61 (0.65–4.02)	0.303	1.54 (0.37–6.34)	0.551
Neurological diseases	2.09 (0.91–4.82)	0.083	1.42 (0.37–5.36)	0.610
Digestive system disease	1.44 (0.75–2.76)	0.278	0.38 (0.09–1.61)	0.187
Cardiovascular disease	1.11 (0.36–3.42)	0.853	0.85 (0.18–4.08)	0.839
15.6-7.4,-1.3498ptKidney/blood/metabolic diseases/other	1.06 (0.44–2.55)	0.901	0.95 (0.24–3.76)	0.941
aCCI risk stratification				
Low risk (aCCI ≤ 3points)	Reference		Reference	
15.6-7.4,-1.3498ptHigh risk (aCCI≥4points)	2.54 (1.07–6.00)	**0.034**	3.03(0.72–12.77)	**0.130**
Combined CHD				
No	Reference		Reference	
15.6-7.4,-1.3498ptYes	2.30 (0.84–6.28)	**0.105**	2.24 (0.45–11.20)	0.328
Albumin stratification				
low level (< 30g/L)	Reference			
normal level (≥30g/L)	0.80 (0.37–1.73)	0.572		
Prealbumin (mg/L)	1.00 (0.993–1.001)	0.264	—	
Lymphocyte count (10^9^/L)	1.24 (0.86–1.79)	0.256	0.67 (0.30–1.52)	0.335
Monocyte count (10^9^/L)	0.38 (0.15–0.96)	**0.04**	1.09 (0.64,1.86)	0.749
Neutrophil count (10^9^/L)	0.98 (0.92–1.04)	0.558	—	
PLR	1.00 (1.00–1.00)	**0.079**	—	
Systemic inflammatory response index	1.02 (0.98–1.06)	0.28	—	
Prognostic nutrition index	1.00 (1.00–1.01)	0.838	1.01 (1.00–1.02)	**0.029**

Continuous variables are presented as median [IQR]. Categorical variables are presented as counts (%). In bold are indicated *P* values considered as statistically significant (*P* < 0.1 or approximately 0.1). BMI, body mass index; APACHE II, acute physiology and chronic health evaluation II; SOFA, sequential organ failure assessment; aCCI, age-adjusted Charlson comorbidity index; CHD, coronary heart disease; PLR, platelet-to-lymphocyte ratio.

In females, BMI (aOR = 0.82, *P* < 0.001) was significantly associated with a decreased odds; NRS 2002 ≥ 4 (aOR = 2.25, *P* = 0.092), aCCI ≥ 4 (aOR = 3.03, *P* = 0.130-a non-significant trend), and prognostic nutrition index (aOR = 1.01, *P* = 0.029) were associated with a increased odds.

### 3.4 Association using restricted cubic spline

**Figure 4:**
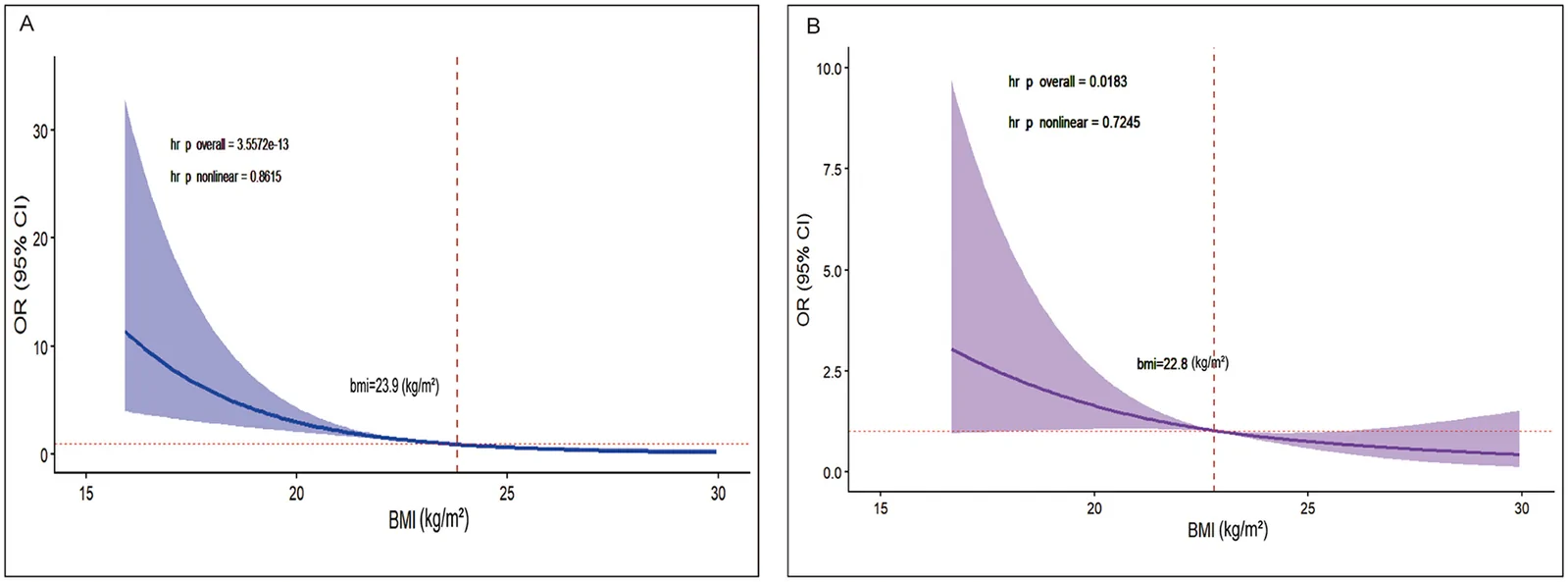
Restricted cubic spline regression analysis (BMI). **(A)** Linear relationship between BMI and pre-existing low muscle mass in males. **(B)** Linear relationship between BMI and pre-existing low muscle mass in females. For A, adjusted age, aCCI, APACHEII score, SOFA score, NRS 2002 score, major disease diagnosis classification, combined CHD, albumin stratification, prealbumin, lymphocyte count, monocyte count, neutrophil count, PLR, systemic inflammatory response index, prognostic nutrition index. For B, adjusted age, aCCI, APACHEII score, SOFA score, NRS 2002 score, major disease diagnosis classification, combined CHD, lymphocyte count, monocyte count, prognostic nutrition index. Likelihood Ratio Test was used in the Restricted cubic spline regression analysis analysis. The solid line represents the estimated odds ratio, and the shaded area indicates the 95% confidence interval.

**Figure 5:**
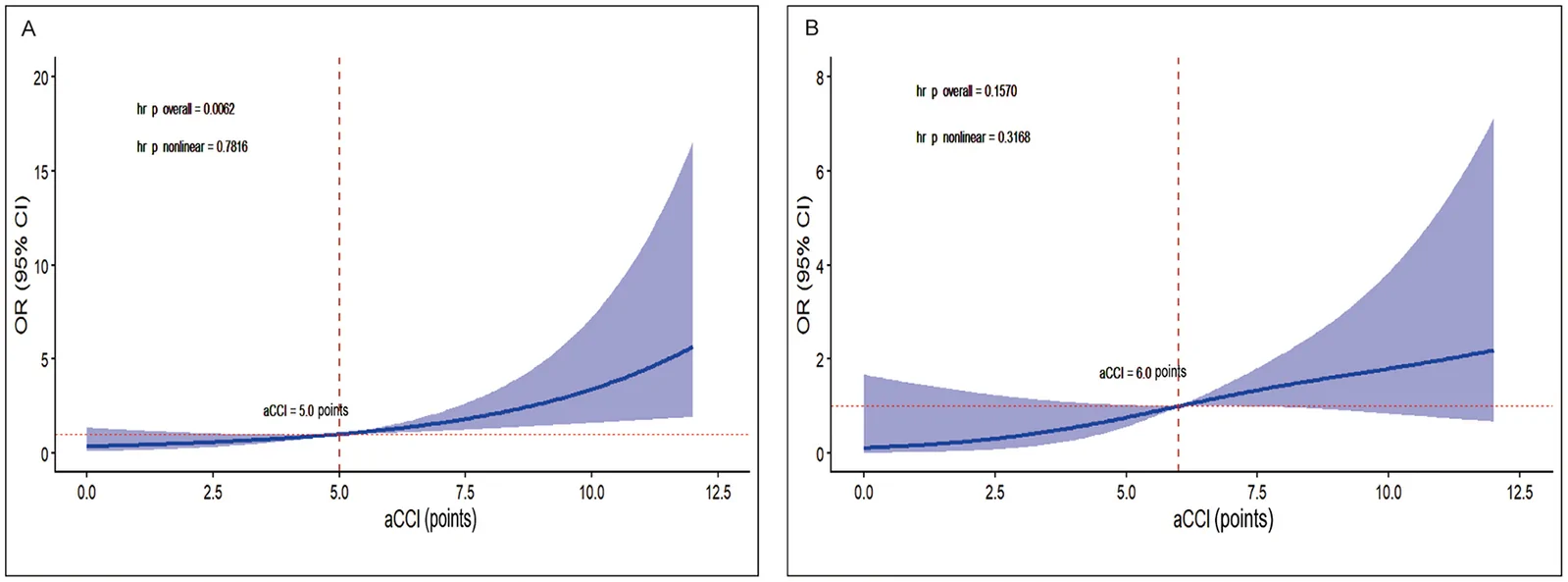
Restricted cubic spline regression analysis (aCCI). **(A)** Linear relationship between aCCI and pre-existing low muscle mass in males. **(B)** No significant relationship between aCCI and pre-existing low muscle mass in females. For A, adjusted age, BMI, APACHEII score, SOFA score, NRS 2002 score, major disease diagnosis classification, combined CHD, albumin stratification, prealbumin, lymphocyte count, monocyte count, neutrophil count, PLR, systemic inflammatory response index, prognostic nutrition index. For B, adjusted age, BMI, APACHEII score, SOFA score, NRS 2002 score, major disease diagnosis classification, combined CHD, lymphocyte count, monocyte count, prognostic nutrition index. Likelihood Ratio Test was used in the Restricted cubic spline regression analysis analysis. The solid line represents the estimated odds ratio, and the shaded area indicates the 95% confidence interval.

RCS showed a linear inverse association between BMI and pre-existing low muscle mass in males (*P* overall = 3.56e−13, *P* non-linear = 0.862). Exploratory visualization suggested a potential plateau in the inverse association at higher BMI levels (Figure 4A). Similar patterns were observed in females (*P* overall = 0.018, *P* non-linear = 0.725), with a similar pattern at higher BMI levels (Figure 4B). Moreover, higher aCCI was linearly associated with higher probability of pre-existing low muscle mass in males (*P* overall = 0.006, *P* non-linear = 0.782), with accelerated rise after aCCI > 5 (Figure 5A). No significant relationship between aCCI and pre-existing low muscle mass in females, shown in Figure 5B. Similarly, higher NRS 2002 scores showed a consistent association with pre-existing low muscle mass in both sexes, with no evidence of nonlinearity in the restricted cubic spline analysis (*P* for non-linearity >0.05) (Supplementary Figure S1). The RCS results of monocyte count and prognostic nutrition index are shown in Supplementary Figures S2, S3.

### 3.5 Association between pre-existing low muscle mass and 28-day mortality

As shown in Supplementary Table S3, there was a significant relationship between pre-existing low muscle mass and 28-days mortality in males (*P* = 0.0007). However, these associations were attenuated and non-significant after adjusting the confounding factors (*P* = 0.83).

## 4 Discussion

This sex-stratified, cross-sectional study of 760 ICU patients reveals four main findings. First, the overall prevalence of pre-existing low muscle mass was 34.0%, substantially higher in males (41.3%) than females (19.8%). Second, higher BMI showed a consistent inverse association with pre-existing low muscle mass. Exploratory RCS analysis suggested that this association may plateau at higher BMI levels; however, the specific values at which this occurs are hypothesis-generating and require external validation. Third, high nutritional risk (NRS 2002 ≥ 4) and high comorbidity burden (aCCI ≥ 4) were independently associated with higher odds of pre-existing low muscle mass in cross-sectional analyses. Fourth, although pre-existing low muscle mass was associated with short-term mortality in unadjusted male analyses, this association was attenuated after comprehensive confounder adjustment, suggesting these factors may mediate the relationship. These findings support early risk stratification but, due to the cross-sectional design, cannot establish causality or directionality.

### 4.1 Higher BMI is inversely associated with pre-existing low muscle mass: exploratory plateau values and clinical implications

In this cross-sectional analysis, the inverse linear relationship between BMI and pre-existing low muscle mass appeared to plateau beyond certain values. Below the exploratory BMI values suggested by the RCS analysis, the probability of pre-existing low muscle mass was higher; above these values, additional BMI was associated with little further decrease in probability. These findings align with the “obesity paradox” literature in critical care, where higher BMI has been associated with improved survival (26, 27). However, our results extend this paradigm by demonstrating that the inverse association between BMI and pre-existing low muscle mass plateaus rather than continues indefinitely. Notably, the exploratory BMI values suggested in our Asian cohort are substantially lower than previously reported cut-offs in Western populations. A study by Bahat et al. (28) using bioimpedance analysis in older adults proposed low muscle mass cut-offs of 1.049 kg/BMI in males and 0.823 kg/BMI in females, which translated to considerably higher BMI equivalents. This discrepancy likely reflects ethnic differences in body composition, as Asian populations typically have lower BMI but higher body fat percentages at equivalent BMI levels compared to Caucasians (29). Furthermore, a United Kingdom Biobank study involving over 50,000 participants identified slow walking pace, multimorbidity, and inflammation as key risk factors for low muscle mass, but did not report BMI-specific thresholds (30). It is critical to emphasize that the BMI plateau values observed in our RCS analysis are purely exploratory and hypothesis-generating. They have not been internally validated (e.g., via bootstrap) nor externally validated in independent ICU cohorts, and should not be interpreted as clinical thresholds or intervention cut-offs. Prospective validation in independent cohorts is required before any clinical application. If validated, these values could help guide risk assessment in ICU populations where rapid nutritional triage is critical.

### 4.2 Nutritional risk and comorbidity burden: sex-specific patterns of association

NRS 2002 ≥ 4 and aCCI ≥ 4 emerged as cross-sectional correlates of pre-existing low muscle mass. In males, NRS 2002 ≥ 4 doubled the odds (aOR = 2.00), and aCCI≥4 was associated with 2.5-fold higher odds (aOR = 2.54). Similar patterns were observed in females, albeit with non-significant trends and wider confidence intervals. The RCS analysis further characterized the relationship between comorbidity burden and pre-existing low muscle mass. In males, higher aCCI showed a linear association with increased probability of low muscle mass (*P* overall = 0.006, *P* non-linear = 0.782), with a notable acceleration in risk when aCCI > 5. The linear nature of this relationship—without evidence of nonlinearity—indicates that even modest increases in comorbidity burden are associated with higher odds of pre-existing low muscle mass. In contrast, no significant relationship between aCCI and pre-existing low muscle mass was observed in females. These findings align with prior literature demonstrating a relationship between comorbidity burden and muscle wasting. A United Kingdom Biobank study by Kiss et al. (30) reported that multimorbidity was significantly associated with higher prevalence of low muscle mass, with prevalence ratios of 1.72 in cancer patients and 1.51 in non-cancer patients. Similarly, a prospective cohort study of ICU patients by Agir and Ateş found a significant negative correlation between Charlson Comorbidity Index scores and rectus femoris muscle thickness (*P* < 0.05) (31). Our study extends these observations by demonstrating that the association is linear and continuous in males with an accelerated rise at aCCI > 5—a potential risk stratification threshold—while showing no significant association in females. This sex-specific pattern underscores the importance of sex-disaggregated analyses in future studies of muscle mass in critically ill populations, as well as personalized management strategies ICU patients. However, given the cross-sectional design, these associations cannot be interpreted as causal; the direction and mechanisms of the aCCI–muscle mass relationship require prospective investigation.

In contrast to the sex-specific pattern observed for comorbidity burden, higher NRS 2002 scores showed a consistent relationship with pre-existing low muscle mass in both sexes. This pattern indicates that nutritional risk, as measured by the NRS 2002 tool, is continuously associated with muscle status at ICU admission regardless of sex: even mild nutritional impairment—not only severe malnutrition—is associated with higher odds of pre-existing low muscle mass. Unlike the aCCI association, which was only significant in males, the NRS 2002 association was consistent across both sexes, suggesting that nutritional risk may be a more universally applicable correlate of low muscle mass than comorbidity burden. These findings support the “nutrition-inflammation-muscle wasting” axis as a plausible biological framework (32). Critically ill patients frequently exhibit a hypercatabolic state characterized by increased protein degradation and negative nitrogen balance. Pre-existing nutritional deficits—captured by higher NRS 2002 scores—likely exacerbate this process by reducing the physiological reserve available to meet the metabolic demands of critical illness. The consistent cross-sectional association we observed across both sexes is consistent with this biological model: greater baseline nutritional impairment may be associated with progressively higher risk of presenting with low muscle mass, regardless of sex. This finding has practical implications: routine NRS 2002 screening upon ICU admission can identify patients across the entire spectrum of nutritional risk, not just those at the extreme, and this approach appears equally informative for both male and female patients. It should be noted that these observations are cross-sectional, and the dose-response pattern does not imply a causal linear relationship.

### 4.3 Sex disparities: implications for personalized management

The marked sex differences observed across multiple analyses warrant careful consideration. First, the prevalence of pre-existing low muscle mass was substantially higher in males (41.3%) than females (19.8%). Second, comorbidity burden (aCCI) showed a linear association with low muscle mass in males (with accelerated rise after aCCI > 5) but no significant relationship in females. Third, the exploratory BMI values from RCS analysis suggested potential plateauing pattern, with sex-specific exploratory thresholds. Fourth, monocyte count was inversely associated with low muscle mass in males only (aOR = 0.38, *P* = 0.04). These sex disparities highlight the need for sex-specific approaches in both research and clinical management of muscle health in critically ill populations.

Several mechanisms may explain these disparities. First, baseline muscle mass is typically higher in males, potentially creating a larger absolute deficit when catabolic processes occur (33). However, this sex difference in absolute muscle mass may be partially offset by differences in CT-defined muscle distribution: males have proportionally greater trunk and upper-body skeletal muscle mass, and the single-slice L3 approach, while validated, may capture different proportions of total muscle mass between sexes (33, 34). Second, the relationship between BMI and skeletal muscle index differs by sex; at equivalent BMI levels, females typically have lower absolute muscle mass but higher relative fat mass, which may influence the BMI–low muscle mass association observed in our RCS analyses (34). Third, hormonal differences, particularly the anti-catabolic and anti-inflammatory effects of estrogen in females, may confer relative protection against muscle proteolysis and comorbidity-induced muscle wasting (35–37). This estrogen-related protection could explain not only the lower prevalence in females but also the absence of a significant aCCI association—suggesting that comorbidity burden may be less detrimental to muscle mass in premenopausal females. However, our study did not collect menopausal status, which represents an unmeasured confounder in the sex-stratified analyses. Fourth, the distribution and severity of specific comorbidities differed between sexes in our cohort, which may contribute to the observed sex-specific aCCI patterns. Finally, the difference in monocyte count between genders suggests that inflammatory pathways may operate differently between sexes (38).

### 4.4 Mortality analysis: low muscle mass as a marker of vulnerability

The pattern observed in our study—significant unadjusted association that attenuated after confounder adjustment—suggests that pre-existing low muscle mass may not be an independent causal driver of short-term mortality, but rather a marker of underlying physiological vulnerability whose mortality effect operates through these pathways. This interpretation does not diminish the clinical importance of pre-existing low muscle mass; rather, it reframes it as a sentinel indicator of overall physiological vulnerability that can guide early risk stratification. This pattern of attenuation is consistent with partial mediation; however, formal mediation analysis with larger sample sizes is needed to confirm this interpretation in future studies.

### 4.5 Limitations

This study has several strengths. The sex-stratified analytic approach acknowledges fundamental biological differences between males and females. The use of restricted cubic splines allowed flexible modeling of nonlinear relationships without arbitrary categorization. The sample size (*n* = 760) offers adequate statistical power for primary analyses.

Several limitations warrant consideration. First, this is a single-center, cross-sectional study, which limits generalizability and precludes causal inference. All associations reported are correlational and cannot establish temporality. And the values suggested by RCS analysis are exploratory, which have not been internally validated (e.g., via bootstrap) nor externally validated in independent ICU cohorts. Therefore, they should not be interpreted as clinical thresholds or intervention cut-offs. Second, our study required an abdominal CT scan at ICU admission for inclusion. Since not all ICU patients undergo abdominal CT imaging, this introduces potential selection bias; patients who receive CT scans may differ systematically from those who do not with respect to clinical characteristics and outcomes. This limitation is inherent to CT-based body composition research in critically ill populations and may affect generalizability. Third, the relatively small sample size in certain subgroups—particularly females (*n* = 247, only 49 with low muscle mass)—resulted in wide confidence intervals for some estimates and limited statistical power for subgroup analyses. Consequently, the female RCS analysis should be interpreted with caution. Fourth, a threshold of *P* < 0.10 was used for statistical significance throughout the analyses, including variable screening and final multivariable models. This liberal threshold was adopted given the exploratory nature of this cross-sectional study and the limited statistical power in certain subgroups. Furthermore, our variable selection followed a simplified implementation of the purposeful selection framework and did not include formal change-in-estimate checks for confounding or systematic re-testing of excluded variables, which may have resulted in residual confounding. Consequently, all findings should be interpreted as hypothesis-generating and require replication in larger, independent cohorts with more conservative significance thresholds. Sixth, despite comprehensive covariate adjustment, residual confounding (e.g., inflammatory cytokines, medication history, pre-ICU physical activity levels) cannot be excluded. Additionally, menopausal status was not collected in our study, which represents an unmeasured confounder in sex-stratified analyses. The estrogen-related protection hypothesis discussed above should therefore be interpreted with caution, as the proportion of premenopausal females in our cohort is unknown.

## 5 Conclusion

Pre-existing low muscle mass affects over one-third of ICU patients, with males disproportionately affected, and is cross-sectionally associated with three modifiable factors routinely assessed at admission: BMI, nutritional risk (NRS 2002), and comorbidity burden (aCCI). Integrating these indicators may enable early risk stratification to guide nutritional and comorbidity management. However, the cross-sectional design precludes causal inference, and the exploratory plateau values require prospective validation. Longitudinal studies with serial muscle assessments are needed to confirm whether targeting these factors can preserve muscle mass and improve outcomes.

## Data Availability

The original contributions presented in the study are included in the article/Supplementary material, further inquiries can be directed to the corresponding authors.
